# An integrated approach to seismic risk assessment using random forest and hierarchical analysis: Pisco, Peru

**DOI:** 10.1016/j.heliyon.2022.e10926

**Published:** 2022-10-07

**Authors:** Luis Izquierdo-Horna, Jose Zevallos, Yustin Yepez

**Affiliations:** Department of Engineering, Universidad Tecnológica del Perú, Lima, Peru

**Keywords:** Analytic hierarchy Process–Saaty, Disaster risk reduction, Hazard, Random forest, Vulnerability, Peru

## Abstract

As Peru is subject to large seismic movements owing to its geographic condition, determining seismic risk levels is a priority task for designing appropriate management plans. These actions become especially relevant when analyzing Pisco, a Peruvian city which has been heavily affected by various seismic events through the years. Hence, this project aims at estimating the associated seismic risk level and its previous requirements, such as hazard and vulnerability. To this end, a hybrid approach of machine learning (i.e., Random Forest) and hierarchical analysis (i.e., the Saaty matrix) was used. Risk levels were calculated through a double-entry table that establishes the relation between hazard and vulnerability levels. Results suggest that the city of Pisco exhibits both medium (lower city areas) and high (higher city areas) hazard levels in similar proportion. In addition, the coast area is considered a very-high hazard zone. Regarding vulnerability, the central area of the city exhibits a medium vulnerability level, whereas the periphery denotes high and very-high vulnerability levels. The interrelation of these components results in overall high-risk levels, with very-high levels in some central areas of the city. Finally, the results from this research study are expected to be useful for the authorities in charge of fostering specific activities in each sector and, simultaneously, as a motivator for future studies within this field.

## Highlights

• Implementation of RF for assessing seismic hazard.
• Implementation of AHP for assessing seismic vulnerability.
• The hazard level in Pisco is similar and constant in the short term.
• High and very-high risk zones have been identified in the city of Pisco.
• Pisco’s seismic risk level is sensitive to the vulnerability of its population.

## Graphical abstract

**Figure:**
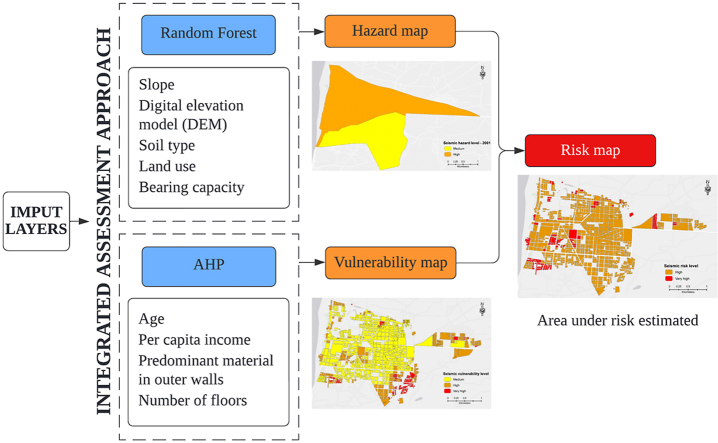

## 1 Introduction

Seismic events are natural phenomena caused by the Earth's inner geodynamics, which are recurrent in some American countries (e.g., Argentina, Bolivia, Canada, Peru, etc.) owing to their geographic location (i.e., the Pacific Ring of Fire) (Tavera and Buforn, 1998). The occurrence of these events leads to material damage, loss of human lives, and the interruption of social, economic and other activities, among others (Lee, 2014). Table 1 denotes information on earthquakes with a magnitude of 7.5 and higher in the Richter scale recorded during the 21st Century in the American continent. This table also denotes losses and damages in the affected regions. According to this record, the earthquakes with the highest magnitude were reported in the United States (2020), 7.8; Mexico (2017), 8.1; Chile (2010), 8.8; and Peru (2001), 8.4. Furthermore, Peru reports highly recurrent seismic activity owing to its location in the subduction of the Nazca oceanic plate and the South American plates (Tavera et al., 2016).

**Table 1:** Record of earthquakes with a magnitude 7.5 and higher and in the Richter scale.

Year	Magnitude	Region	Country	Consequences of the event	Source
2021	7.5	Loreto	Peru	1 dead, 17 injured, and 5689 homes and buildings damaged	(USGS, 2022)
2020	7.8	Alaska	United States	A few homes and buildings damaged	(USGS, 2022)
2019	8.0	Loreto	Peru	2 dead, 30 injured, and 1010 homes and buildings damaged	(USGS, 2022)
2019	7.5	Pastaza	Ecuador	1 dead, 9 injured, and 22 homes and buildings damaged	(USGS, 2022)
2017	8.1	Chiapas	Mexico	94 dead and 250 injured	(GFZ, 2022)
2016	7.8	Esmeraldas	Ecuador	668 dead, 27,735 injured, and 7000 homes and buildings destroyed	(USGS, 2022)
2014	8.2	Tarapacá	Chile	6 dead and 1 damaged building	(Hayes et al., 2017)
2010	8.8	Bio-Bio	Chile	523 dead, 12,000 injured, and 374,092 homes and buildings damaged/destroyed	(Hayes et al., 2017)
2007	8.0	Ica	Peru	514 dead, 1090 injured, and 39,700 homes and buildings damaged	(Hayes et al., 2017)
2005	7.5	Loreto	Peru	5 dead, 60 injured, and 200 homes and buildings damaged	(Hayes et al., 2017)
2001	8.4	Arequipa	Peru	74 dead, 2689 injured, 35,601 homes and buildings damaged, and 17,584 homes and buildings destroyed	(Hayes et al., 2017)
2001	7.6	Moquegua	Peru	1 dead, 30 injured, and 100 homes and buildings destroyed	(Hayes et al., 2017)

In Peru, some areas have reported seismic events more frequently than the rest of the country. For instance, the departments with frequent earthquakes over a magnitude of 5.0 in the Richter scale are Arequipa (16 events), Ica (12 events), Tacna (9 events) and Ucayali (8 events) (Hayes et al., 2017; Tavera et al., 2016). Specifically, some cities in Ica are among the most affected by these events (e.g., Chincha, Ica and Pisco) (Elnashai et al., 2008). In fact, one of the most important occurrences took place in Pisco on August 15th, 2007 (8.0 Mw) (Hayes et al., 2017). According to Elnashai et al. (2008), this earthquake left 600 dead, several hundred injured, 50,000 buildings destroyed, and more than 20,000 buildings damaged. However, the Instituto Nacional de Defensa Civil (INDECI) (2008) reported a 7.9 Mw magnitude, leaving 519 deaths and 1844 injured, and affecting 60% of the city structures (55,010 destroyed and 21,583 damaged). However, Tavera and Bernal (2008) state that the same earthquake affected 80% of all local structures (230,000 damaged and 52,150 destroyed), and left 595 dead and 318 people missing. D'Ercole et al. (2009) state that this earthquake had a magnitude of 8.1 Mw, left 596 deaths and 1292 injured, and affected 192,700 homes and buildings. Besides the different estimates reported, these data evidence the importance of implementing seismic risk mitigation plans and fostering strategies aimed at building resilient communities.

According to the United Nations (UN) (2006), risk is the probability of harmful consequences, or expected loss of lives, people injured, property, livelihoods, economic activity disrupted (or environment damaged) resulting from interactions between natural or human-induced hazards and vulnerability conditions. In this sense, the Centro Nacional de Estimación, Prevención y Reducción del Riesgo de Desastres (CENEPRED) aiming at finding probable effects and social, economic and environmental consequences for a community, determines that risk is the result of relating hazard with the vulnerability of said elements (CENEPRED, 2014). In addition, INDECI (2001) states that risk is the result of assessing the impacts of the hazards that an element is exposed to, and their degree of vulnerability. This proves the need for a joint analysis of hazard and vulnerability to obtain consistent results that provide valuable information in the decision-making process (Koks et al., 2015).

Hence, aiming at assessing the risk level, several studies were performed implementing different techniques. For example, in Aceh (Indonesia), risk was estimated integrating hierarchical assessment techniques (e.g., Hierarchical Cluster Analysis (HCA) and Analytic Hierarchy Process (AHP)) with machine learning methods (e.g., neuronal networks, Random Forest). Here, the former measure vulnerability, whereas the latter assess probabilistic risk (Jena et al., 2020a, Jena et al., 2020b). This hybrid risk analysis model is flexible and reliable since it integrates both analysis techniques (Yariyan et al., 2020). Currently, machine learning (i.e., neuronal networks, random forest, regressions) has become popular as a probabilistic model building tool (Jena et al., 2020a, Jena et al., 2020b). Simultaneously, these studies are complemented by a Geographical Information System (GIS) environment. For example, in Coimbra (Portugal) seismic risk was assessed by systematizing building characteristics as qualitative information (e.g., structure building system, irregularities and interaction, among others) integrating them in a GIS platform, and quickly identifying specific characteristics of the study area (Vicente et al., 2011), as well as people and buildings at risk to implement specific impact mitigation strategies (Yariyan et al., 2020).

Nevertheless, risk cannot be established without prior determination of hazard and vulnerability. On one hand, to determine hazard, the occurrence of a phenomena must be estimated based on its origin, records through time and location (CENEPRED, 2014). In this sense, the Centro Peruano Japonés de Investigaciones Sísmicas y Mitigación de Desastres (CISMID) (2017) states that, to define seismic hazard, we must consider variables associated to the event and study area (e.g., seismic demand acceleration, speed of movement, predominant material). For example, some studies assess seismic hazard via machine learning (i.e., neuronal networks) using parameters such as Peak Ground Acceleration (PGA), intensity variation, slope, distance from fault lines, epicenter density, among others (Jena et al., 2020a, Jena et al., 2020b). Seismic hazard can also be assessed through a hierarchical process oriented toward GIS using geological, geodesic, geotechnical and geophysical parameters (Karimzadeh et al., 2014), or information related to area characteristics (e.g., past earthquake epicenters, active fault lines) (Ahmad et al., 2017; Funning et al., 2005). Another widely used alternative for assessing hazard level is using satellite images (Maruyama et al., 2012), and information from surveys performed at the study area (Matsuoka et al., 2014).

However, to assess vulnerability, we need to use practical tools that integrate quantitative and qualitative criteria with wider assessment approaches to provide results with a higher validity level (Birkmann and Wisner, 2006). The above mentioned approaches must guarantee a transversal vision of the study area, so once they are integrated to the action plans, they can contribute to mitigating damages after a disaster occurs (Cutter et al., 2003; Izquierdo-Horna and Kahhat, 2020). A representation of the study area can be performed through physical, social, economic and environmental indicators (United Nations, 2005). In this sense, indicators based on geographical environment, preexisting social, economic and political conditions, exposure degree, among others, were used as vulnerability analysis factors (Rufat et al., 2015). Depending on information availability, certain indicators and methods are more relevant than others. For example, in Iran (S. Lee et al., 2019) and Malaysia (Suhaiza Sauti et al., 2020), machine learning techniques (e.g., neuronal networks) were implemented in GIS environments for assessing seismic vulnerability. Similarly, in Portugal (Fernandez et al., 2016) and Romania (Albulescu et al., 2020), seismic vulnerability was analyzed via social and physical factors through a multicriteria decision analysis.

This research study integrates assessment methods based on machine learning (i.e., Random Forest) and hierarchical methods (i.e., AHP) for assessing seismic risk. The first will assess hazard in several hypothetical scenarios and predict their associated level through a collection of independent, random vectors distributed identically in a tree structure (Breiman, 2001), wherein one single vote will be casted to precisely determine its importance (Cutler et al., 2007). The latter are used to assign numerical values to what are essentially abstract concepts in order to create easy-to-interpret instruments (Saaty, 1988). The vulnerability analysis shall follow the proposal by Izquierdo-Horna and Kahhat (2020), a bidimensional, social and physical analysis. As a methodological implementation, the city of Pisco (Peru) was selected due to its history of earthquakes of significant magnitude (Hayes et al., 2017), and its propensity to new high-magnitude events (Tavera et al., 2016). The application of these results is expected to contribute positively to disaster risk management in the region.

## 2 Methodological framework

**Figure 1:**
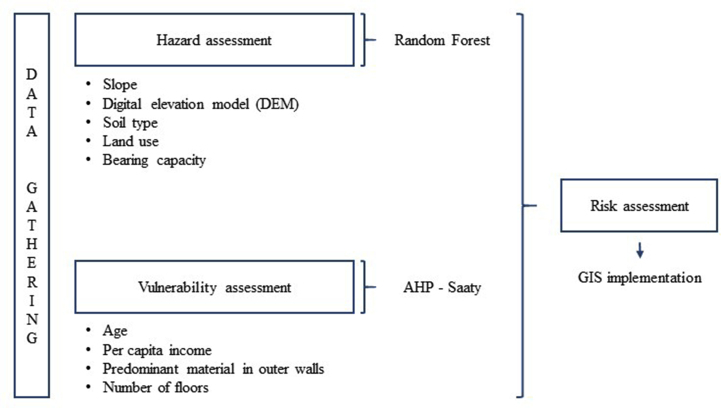
Methodological framework – GIS: Geographical Information System.

Aiming at determining the seismic risk level in the city of Pisco, Figure 1 denotes the methodological framework implemented for this research.

### 2.1 Case study information

#### 2.1.1 Study area

The city of Pisco is located in the Peruvian central coast, at 13°42′35″S, 76°12′11″O. It is the capital of the Pisco province and part of the Ica region, and its population is approximately 67,467 (INEI, 2018). Pisco is one of the Peruvian cities that is most affected by earthquakes owing to their high recurrence and magnitude. This is largely owing to the city's geographical location as it is situated on fluvial deposits that belong to the old alluvial fan of the Pisco river, so its surface is covered by silty sand and poorly graded gravel (INDECI, 2008). The city is also exposed to soil liquefaction, loss of bearing capacity and flooding owing to tsunamis (INDECI, 2008).

#### 2.1.2 Data acquisition

**Figure 2:**
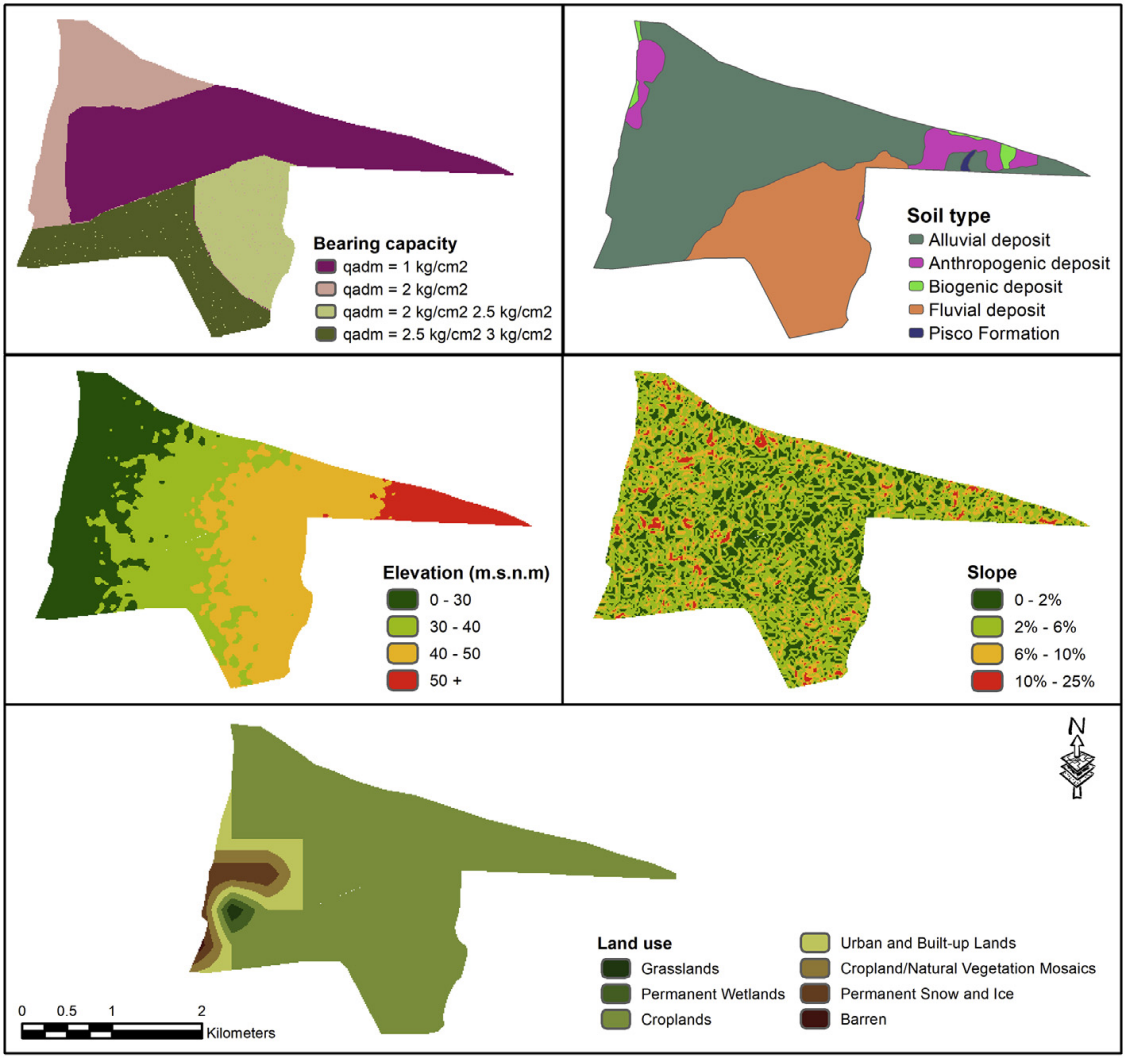
Parameters used for analyzing seismic hazard.

This research project uses several free access data sources for analyzing seismic risk. On one hand, for hazard assessment, parameters, such as slope, digital elevation model (DEM), soil type, land use, and bearing capacity were considered. DEM was obtained from shuttle radar topography mission (SRTM) (https://srtm.csi.cgiar.org/srtmdata/) with a spatial resolution of 30 m × 30 m. Then, slope spatial distribution was estimated based on DEM. Soil types were obtained from the Geocatmin site (https://geocatmin.ingemmet.gob.pe/geocatmin/). Land use is a MODIS MCD12Q1 product with a spatial resolution of 500 m × 500 m (https://earthexplorer.usgs.gov/). Finally, bearing capacity and data required for model implementation were obtained from INDECI (2001) and CISMID (2011). Figure 2 denotes the parameters used in this study.

On the other hand, for assessing vulnerability, considering its bidimensional analysis (i.e., social and physical), and the availability of information, the following parameters were considered: age, per capita income, predominant material in outer building walls, and number of floors. To this end, images from the National Georeferenced Data Platform (GEO Peru, https://visor.geoperu.gob.pe/) were used together with microdata from censuses and surveys obtained from the Instituto Nacional de Estadística e Informática (INEI, http://iinei.inei.gob.pe/microdatos/) and microzonification maps provided by CISMID (2012). Finally, this information was processed and incorporated for analysis in the QGis 3.16 software, obtaining information layers according to the required descriptors. Table 2 below denotes the parameters used in this research.

**Table 2:** Parameters used for analyzing seismic vulnerability.

Dimension	Variable	Descriptor
Social	**Age (years old)**	30 to 49
		18 to 29
		13 to 18 and 50 to 59
		4 to 12 and 60 to 64
		0 to 3 and over 65
	**Per capita income (USD)**	Over USD 1000
		USD 700–1000
		USD 300–700
		Minimum salary, USD 300
		Under USD 300
Physical	**Predominant material in outer walls**	Brick
		Wood
		Adobe
		Quincha
		Other precarious materials
	**Number of floors**	1
		2
		3
		4
		5 or more

### 2.2 Integrated approach: random forest and AHP–Saaty

To determine the seismic risk level, we proposed a methodology that combines machine learning with hierarchic learning techniques. In this sense, to determine seismic hazard level, Random Forest (RF) will be used because it is efficient in finding frequent, nonlinear patterns in many classification problems, and, at the same time, facilitates working with numerical and categorical data without requiring additional treatments (e.g., normalization of numerical data) (Breiman, 2001; Lawler et al., 2006).

However, an AHP–Saaty will be applied to determine the corresponding vulnerability level. One of the advantages of this hierarchical process is that it solves diverse criteria problems with multiple values (Mendoza et al., 2019). It is also the method proposed by the executing public body, CENEPRED, subscribed to the Peruvian Defense Ministry, to solve complex problems through a visually structured model, which facilitates the identification and weighing of decision-making parameters (CENEPRED, 2014).

#### 2.2.1 RF: considerations and assumptions

RF is a supervised machine learning algorithm that can be used for classification or regression. It is a collection of decision trees, in which each tree is trained with a random subsample replacing the original sample (bootstrapping) and a subsample of available predictors. The final prediction is the result of the majority of the votes from the predictions of each trained tree (Cutler et al., 2007). This modeling diagram reduces the possibility of overtraining, since trees that form the scheme have low correlations and high variance (Costa et al., 2018).

#### 2.2.2 AHP–Saaty: considerations and assumptions

The Saaty AHP is a multicriteria technique used for making decisions that identifies and organizes goals within a hierarchy, and fosters pair comparisons among the relevant elements being compared (Saaty, 1988). For their implementation, we first arrange the descriptors in hierarchical order from the category assessed. Then, these descriptors are categorized according to the assessment scale to build value judgment and normalization matrixes. Finally, priority and consistency vectors are calculated to interpret results (Mendoza et al., 2019).

### 2.3 Hazard, vulnerability and risk assessment

#### 2.3.1 Seismic hazard assessment

For this study, hazard is defined as the probability that a natural phenomenon occurs in a specific place within a determined time, with a certain intensity and defined frequency (CENEPRED, 2014). As the last precedent of this assessment in Pisco, in 2001 INDECI performed a seismic hazard assessment identifying four hazard levels (i.e., very high, high, medium and low) (INDECI, 2001); however, for the purposes here, we considered the more representative hazards in the area since there was more information available on their predictors. Site conditions used to characterize hazard levels do not remain constant as they experiment modifications after each seismic event (INDECI, 2008).

To assess the seismic hazard level for different scenarios, we developed and validated a RF model with the following predicting variables: soil type, DEM, land use, land slope and bearing capacity. The predicted variable was the hazard level recorded in 2001. Since this data had a different spatial resolution, 10 m × 10 m maps were resampled. To avoid overcompensation, we divided the data in 80% to train the model and 20% to test it. RF hyperparameters were optimized by a randomized grid search with crossed validations. Optimization refined the number of trees (estimators) and the maximum depth for each tree. The former facilitate variability and decorrelation among trees, whereas the latter reduce the possible overcompensation in each trained tree (Zevallos and Lavado-Casimiro, 2022). The optimized metric is accuracy, using a 5-fold crossed validation.

As a result of total hyperparameter optimization, 250 classification trees were built using Gini criteria with a maximum depth of 10. To assess the model's performance, we used the 20% data we had previously separated to build a confusion matrix to check the accuracy and classification mistakes of the model.

#### 2.3.2 Seismic vulnerability assessment

Considering that the assessment of seismic vulnerability requires not only quantitative approaches but also qualitative approximations (Birkmann and Wisner, 2006), some researchers state that an adequate way to assess vulnerability is via social, demographical and socioeconomic factors (Cutter et al., 2003). This research will be guided by the analysis proposed by Izquierdo-Horna and Kahhat (2020) since they promote the need of a comprehensive approach via a bidimensional analysis wherein the physical dimension (i.e., built environment) is as relevant as the social dimension (i.e., final user). Here, social characteristics (e.g., age and per capita income) tend to change more rapidly over time than physical characteristics (e.g., predominant material in outer building walls and number of floors).

To treat data and build hierarchically, we followed the recommendations by CENEPRED (2014). This technique determines the importance of the selected parameters according to the Saaty scale by pair comparison in a 4 × 4 matrix. Therefore, rows are compared against columns. In addition, the matrix diagonal will be equal to 1 since it is a parameter of equal importance. Then, the normalization matrix is defined; it denotes the prioritization vector given by the weight related to the parameters being analyzed. Finally, the consistency ratio is calculated. Its value must be under 10% (RC < 0.1) to guarantee an acceptable consistency level in pair comparison. If this value is not achieved; the matrix criteria must be reassessed. We also considered a random index of 0.882 because this is a matrix that depends on four parameters in comparison to the 100,000 same-order matrixes simulated (Aguarón and Moreno-Jiménez, 2003).

#### 2.3.3 Seismic risk assessment: holistic perspective

Seismic risk analysis corresponds to an analysis of hazard probability and vulnerability levels of the element exposed within a determined area (INDECI, 2006); however, CENEPRED (2014) states that it is possible to assess the risk level via a double-entry matrix: a hazard level matrix and a vulnerability level matrix. To understand this graphically, we will analyze a matrix wherein, to estimate risk, we intersect (e.g., Cartesian plane) the Y axis (hazard levels) and the X axis (vulnerability levels).

For assessing the accuracy in the risk map, we performed a visual identification of the area to study via Google Street View. The identification consisted in revising areas with high and moderate risk level and verifying the structure quality of the surrounding buildings.

## 3 Results

### 3.1 Seismic hazard level

From the obtained results, we can see that the most relevant parameter identified in the implemented model is the soil type with 62.3%, while the parameter with the least impact on the classifications made is land use with less than 1% (Table 3). Bearing capacity has a 4.5% importance in the general classification.

**Table 3:** Feature of importance for RF.

Variable	Importance
Soil type	0.623
DEM	0.301
Bearing capacity	0.045
Slope	0.027
Land use	0.004

**Figure 3:**
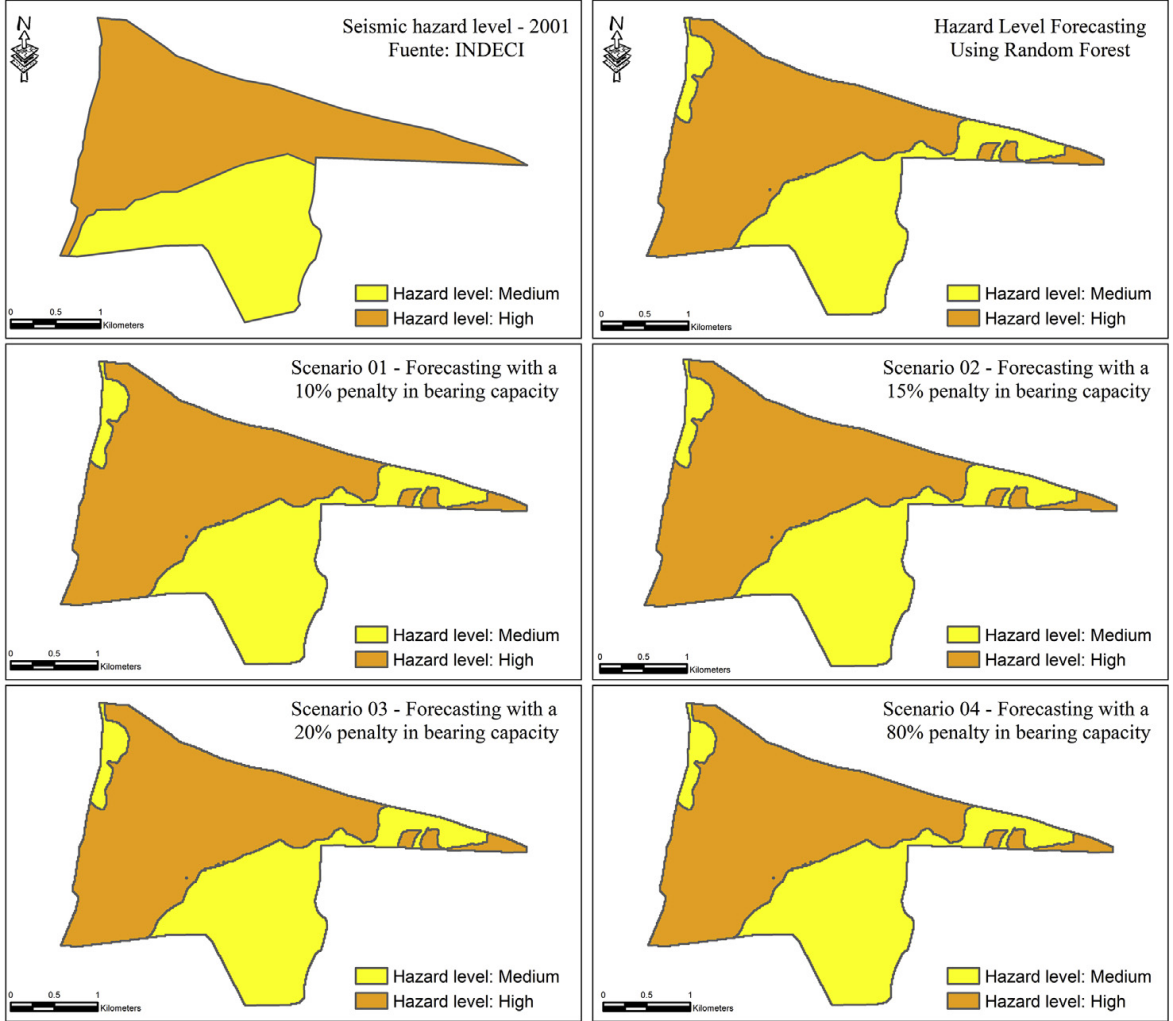
Reproduced hazard level for 2001 and predicted levels for different scenarios of bearing capacity.

Figure 3 denotes spatial distribution for the 2001 situation and the four scenarios built. There are some erroneous zones that indicate a moderate level of hazard to the northwest and west of the map, instead of a high level. Scenarios considered were predicted by penalizing the bearing capacity predictor because out of all factors used, this is the one that may change over the years with each event occurrence. Assigned penalizations were 20%, 80%, 85% and 90% of the values reported in 2001. As it may be observed, there is no substantial difference among the results for the different scenarios. This is likely because bearing capacity only has a 4.5% influence in hazard classifications.

### 3.2 Seismic vulnerability level

**Figure 4:**
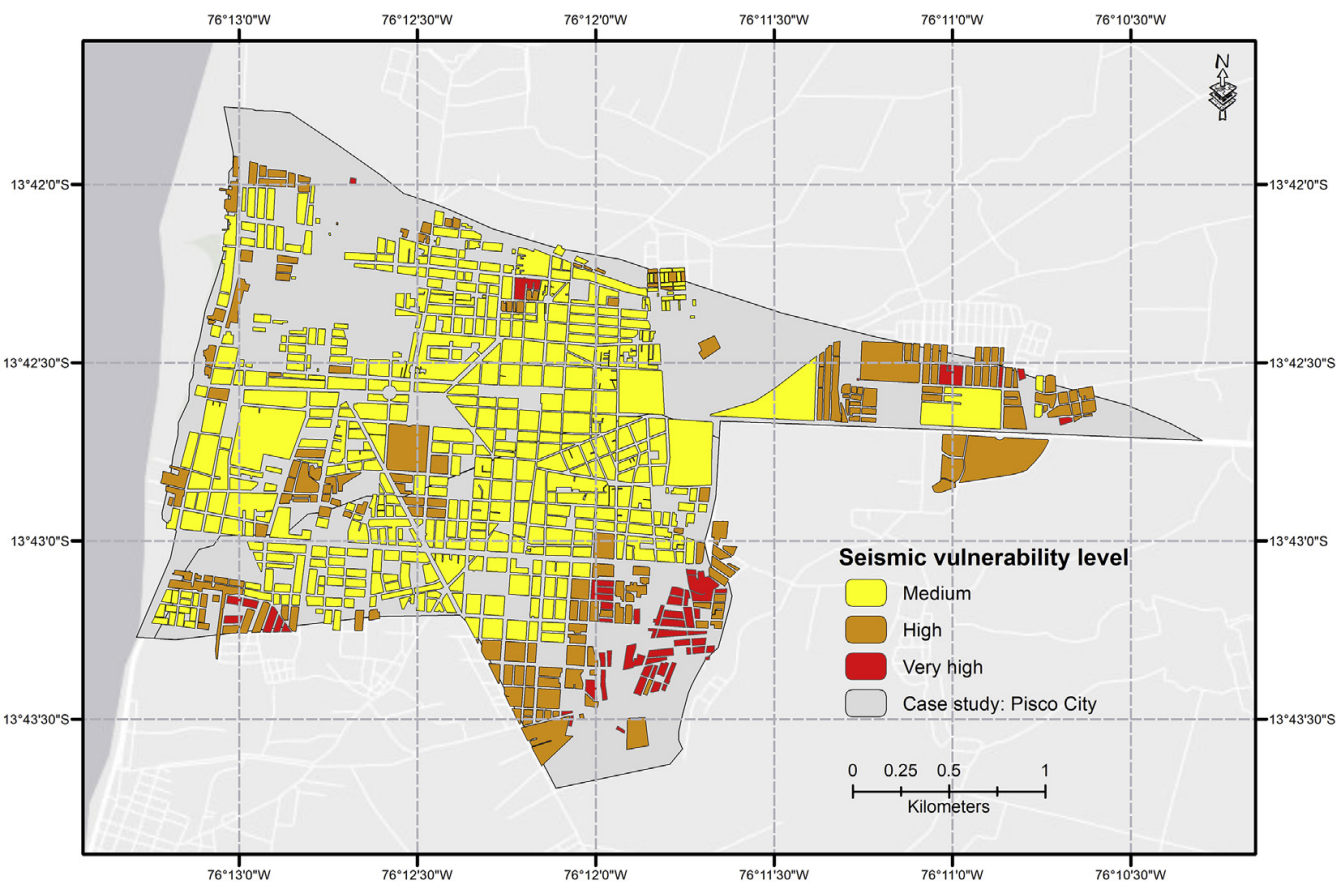
Seismic vulnerability map for Pisco.

After performing the hierarchical analysis with the relevant parameters, we georeferenced using block to be able to visualize the distribution of vulnerability in the terrain. Hence, we obtained the earthquake vulnerability map for the city of Pisco (Figure 4), which evidences three out of the four levels considered: medium, high, and very high. Here zones with high and very-high vulnerability are in the district periphery. As for the social dimension, INEI (2018) points out that the elder population has increased considerably in the city of Pisco: in 1993, they represented 5.0%; in 2007, 6.8%; and in 2017, 8.2%. The population under 15 represented 28.8% of the whole population in 2017. However, the population percentage in medium, medium-low and low socioeconomic layers diminished: in 2013, they represented 83.30% and, in 2017, 58.40% reported less than USD 300 in income (Ministerio de Trabajo y Promoción del Empleo, 2022), which is considered very low according to minimum life conditions. Regarding the physical dimension, INEI (2018) states that, in 2017, 25.49% of all existing homes and buildings were made with precarious materials (e.g., adobe, quincha, plywood, straw mat). Moreover, according to the micro-zonification map issued by CISMID (2012), around 40% of buildings (regardless of number of floors) are not in good conditions owing to lack of technical supervision before and after its construction or an earthquake event. However, these buildings must be considered since 81% are used as homes and 8.1% as educational centers.

### 3.3 Seismic risk level

**Figure 5:**
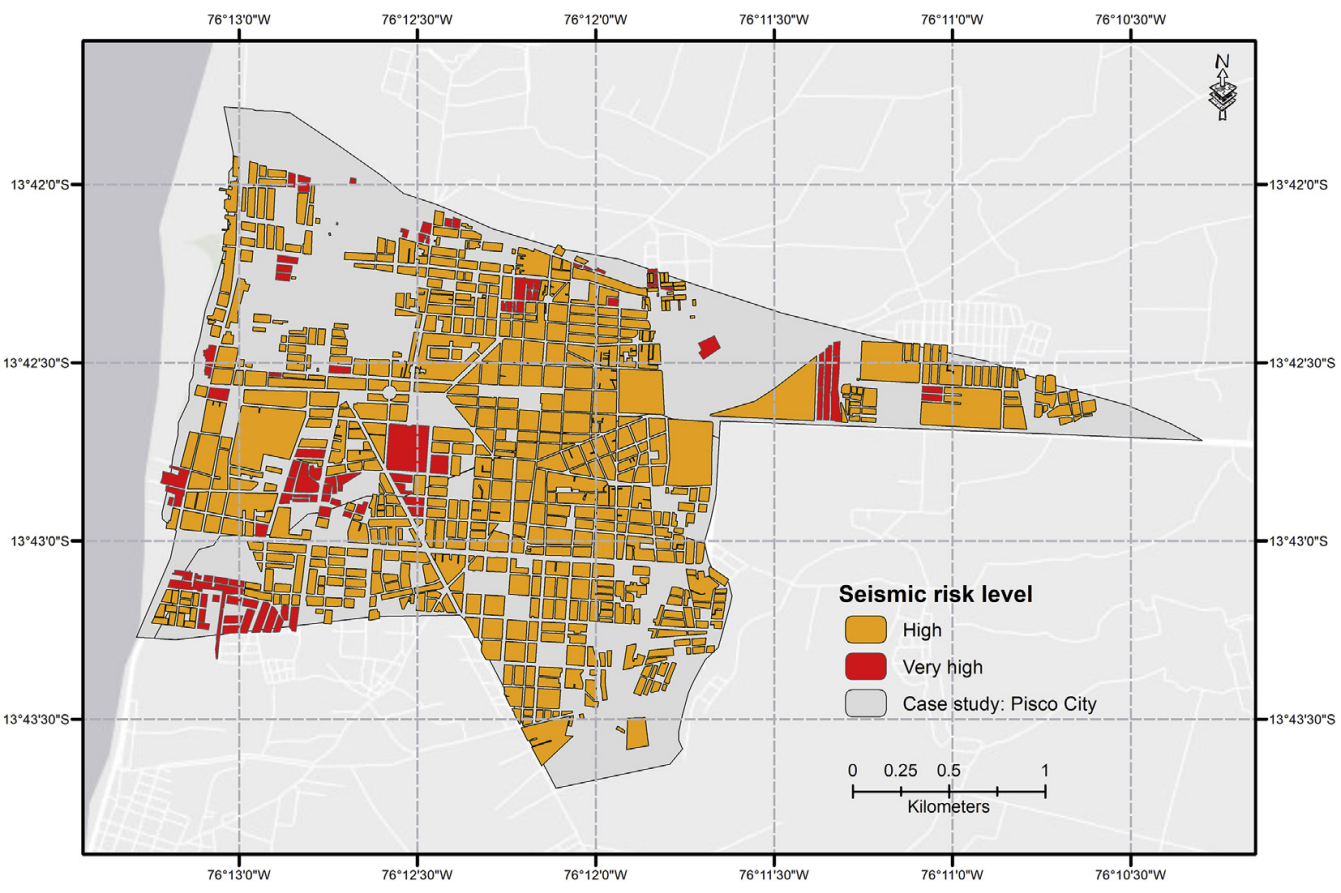
Seismic risk map for Pisco.

Finally, after the hazard and vulnerability analysis in the study area was finished, seismic risk level was determined using the double-entry table (CENEPRED, 2014). Based on the risk levels defined, the risk level map was drafted, denoting high and very-high levels (Figure 5). The identification of these risk levels facilitates the structure and design of focalized plans aimed at avoiding damage and loss. Likewise, being able to locate them spatially fosters the proposal of structural and non-structural risk mitigating solutions, such as injections via compaction to deeply densify mainly sandy soils, thus preventing the liquefaction effect. Moreover, we noted that very-high risk zones are commonly further away from the city center and closer to the coast. However, via visual inspection, we analyzed some sectors identified as very-high risk using Google Street View in order to further validate the results.

## 4 Discussion

The seismic risk level identified in Pisco based on the information collected was high and very high. These levels suggest that it is a priority action area for implementing strategies to mitigate risk and promoting the construction of resilient cities. However, development to this date is scarce. This research used a machine learning and a hierarchical analysis approach to assess hazard and vulnerability levels, respectively. For the former, hazard level was built based on the information proposed by INDECI (2001). Due to geographic location and terrain distribution, the coast has not been added in the analysis since it is an area with inadequate construction characteristics (e.g., low bearing capacity, prone to liquefaction) (INDECI, 2008).

On the other hand, it can also be observed that the scenarios generated from the variation in bearing capacity maintain a fairly constant hazard (Figure 3). This is probably due to the fact that bearing capacity has only a 4.5% influence on the hazard level classifications. Furthermore, we note that the seismic hazard level predicted for the year 2001 is very similar to the soil type map, with few differences probably caused by the influence of the other most important variable: elevation (Figure 2). Likewise, it is important to mention that in spite of all the variability contributed by slope, this factor is not very relevant and has a 2.7% importance in the predictions, causing that its variability is not reflected when classifying the level of seismic hazard. Thus, it can be inferred that the level of seismic hazard remains almost constant in short periods of time considering that other scenarios with variations of other predictors are less probable since the time horizons are shorter: a change in the composition of the soil type or a modification of the topography and slope would require the passing of geologic time.

However, the vulnerability results obtained were compared to the studies performed by the Science and Technology Research Partnership for Sustainable Development (SATREPS), which is sponsored by the Japan Science and Technology Agency (JST) and the Japan International Cooperation Agency (JICA). These previous studies mainly consist of assessing area weaknesses by simulating its seismic response and damage level (Zavala et al., 2011), monitoring maximum resilience between 2007 and 2011 (Hoshi et al., 2014), and assessing resilience after urban recovery processes through field interviews (Murao et al., 2013). Still, these studies were based on information after the 2007 earthquake, and therefore, when comparing said results against the results obtained in this research (whose source is the 2017 census), certain differences arise.

The most notable change was found in the city center. Previous studies state that the most vulnerable areas were those closer to downtown; however, this research study found the opposite–vulnerable areas were further away at the periphery. Still, this difference can be explained: in the past, homes and buildings were located in the downtown area and, for the most part, built with precarious materials; dwelling density was also lower than currently. Hence, the time variation in sources of available information becomes a relevant factor for the identification process.

The importance of this research stems from the constructed environment–final user concept; that is, the growth level experienced in Pisco during the last 15 years denotes how variable and dynamic this area is. Such disorderly growth is coupled with an increase in population density and resource demand. The changes in its constructed area and social characteristics (e.g., age, economic income, etc.) make it necessary to constantly update the level of exposure and degree of potential losses caused by the occurrence of an event. On the other hand, having current and reliable information that reflects the study case situation gives authorities, and those with decision-making power, the possibility to better assign resources and foster resilience in areas that are potentially more affected.

Finally, one of the most relevant limitations for the design and implementation of this methodological proposal lies in the absence of continuous measurements for the case study (e.g., soil studies, surveys, etc.). This is due to the fact that the time lag between current conditions and the reported reference conditions is greater than 5 years approximately, which has repercussions not only on urban development and territorial planning, but also on the livelihoods and living conditions of the population.

## 5 Conclusions

The results from this research suggest high and very-high risk areas distributed throughout the Pisco territory. These results are coherent with the city's precedents, the anthropogenic characteristics of the population, and the level of exposure to seismic events. Hazard, vulnerability, and risk maps may be considered as reference instruments to design strategies aimed at preventing losses. The methods implemented to design these maps revealed an accuracy level of 0.99 for RF and consistency indexes lower than 0.04 for hierarchical analysis. The parameters that support these results refer to the most determining parameters for the area and were selected by their intrinsic characteristics and based on literature reviews. This study applied RF to classify hazard in the year 2001, and then to project hazard scenarios changing bearing capacity conditions. For vulnerability, we used AHP. One of the most relevant limitations for implementing these models was the number of observations, and the availability and quality of information.

This project used open access data and official government sources, which allowed for rapid validation and implementation. Some data clusters were compiled from the USGS, GEOCATMIN, GEO PERU, INEI and SIGRID platforms. The project also includes the implementation of a GIS system, which makes result visualization and communication more efficient. As these data clusters provided the information required for hazard characterization, the factors considered for these purposes were DEM, slope, land use, soil type and bearing capacity. Likewise, vulnerability is characterized by age, socioeconomic level, predominant construction material used in outer walls, and number of floors. Hence, depending on information access, we can work with additional parameters such as liquefaction, fault lines, etc. Still, hazard remained almost constant since relevant changes in stratigraphy and topography are related to geological time variations. Similarly, vulnerability may also be supplemented with information on education, access to health insurance, access to basic services, etc. In consequence, the validity, adequacy, and prediction or classification capacity of each parameter will largely depend on the study approach used and the type of research performed.

Finally, considering that the source of information used for the vulnerability analysis is dated 2017 and, as a reference, one of the relevant studies for soil characterization of Pisco dates back to 1999 it is to be expected that the results obtained have associated uncertainties. This scenario leads to limited applicability given that the input information presents a time lag with the current time; however, we consider that as a methodological proposal, this study is representative and can be validated through visual inspections of the sector. Thus rendering this methodology reproducible if considering the corresponding assumptions against conditioning, triggering and anthropogenic factors. We hope this approach contributes to prospective risk management and facilitates an optimal design of mitigation and emergency response plans. We also hope that this study is complemented by the implementation of more efficient auditing methods that may restrict empirical self-building activities within the assessed area.

## Declarations

### Author contribution statement

Luis Izquierdo-Horna: Conceived and designed the experiments; Analyzed and interpreted the data; Wrote the paper.

Jose Zevallos: Performed the experiments; Analyzed and interpreted the data.

Yustin Yepez - Analyzed and interpreted the data; Contributed analysis tools or data; Wrote the paper.

### Funding statement

This work was supported by Universidad Tecnológica del Perú, within the framework of the “Research Projects I+D+i 2021- 1” agreement [P2021-LIM04].

### Data availability statement

Data are available on reasonable request.

### Declaration of interest’s statement

The authors declare no conflict of interest.

### Additional information

No additional information is available for this paper.
